# The complete chloroplast genome of *Rhus punjabensis* var. *sinica*

**DOI:** 10.1080/23802359.2021.1925983

**Published:** 2022-01-24

**Authors:** Yunjia Pan, Jinglu Feng, Yulin Lin, Hui Yao

**Affiliations:** a Institute of Medicinal Plant Development, Chinese Academy of Medical Sciences, Peking Union Medical College, Beijing, PR China; b Engineering Research Center of Chinese Medicine Resource, Ministry of Education, Beijing, PR China

**Keywords:** *Rhus punjabensis* var. sinica, chloroplast, genome sequence

## Abstract

*Rhus punjabensis* var. *sinica* belongs to the family Anacardiaceae in the order Sapindales. In this study, we first reported the complete chloroplast genome sequence of *R. punjabensis* var. *sinica*. The cp genome was sequenced on Illumina Hiseq 2000 platform. The sequence was found to be 159,617 bp in length with 37.9% GC contents, including a large single-copy region of 87,694 bp, a small single-copy region of 18,971 bp, and a pair of inverted repeats of 26,476 bp. The chloroplast genome of *R. punjabensis* var. *sinica* contains 133 genes, including 86 protein-coding genes, 8 rRNA genes, and 2 pseudogenes identified by CPGAVAS2 and BLAST search, and 37 tRNA genes annotated by tRNAscan-SE. Maximum-likelihood (ML) phylogenetic analysis showed that *R. punjabensis* var. *sinica* was sister to *Rhus potaninii*.

*Rhus punjabensis* var. *sinica*, belonging to the family Anacardiaceae in the order Sapindales, grows on hill and mountain forests at an altitude of 400−3000 m. Galla Chinensis, a natural traditional Chinese medicine, is formed by *Rhus* gall aphids that live on the leaves, petioles, and wings of the primary host plants *Rhus* (Ren et al. 2017), and is widely used in China (Zhang et al. 2015). The host plants include *Rhus chinensis*, *Rhus potaninii*, *R. punjabensis* var. *sinica*, *Rhus typhina*, and *Rhus glabra*. The main component of Galla Chinensis is tannic acid, which has antioxidation effect (Tajima et al. 2016), antidiarrheal effect (Yang et al. 2017), analgesic and anti-inflammatory effects (Sun et al. 2018). Because there are many researches related to pharmacology but less on its genome, this study provides a theoretical basis for the phylogenetic relationship of *Rhus* and the coevolution between host trees and *Rhus* gall aphids.

The specimen was stored in Herbarium of Institute of Medicinal Plant Development (voucher: Pan0102). Fresh leaves of *R. punjabensis* var. *sinica* were collected from Enshi City, Hubei Province (29°44′02″N, 109°29′48″E) at an altitude of 600 m on 5 September 2019. Its total genomic DNA was extracted using QIAGEN DNeasy Plant Mini Kit (QIAGEN, Hilden, Germany). The whole genome was sequenced on Illumina Hiseq 2000 platform (Illumina, San Diego, CA), and 8.12 G data were acquired. Clean data were further assembled into a complete chloroplast genome using SOAPdenovo version 2 (Hong Kong, China) (Luo et al. 2012) and SSPACE (Boetzer et al. 2011). The protein-coding genes, rRNA genes, and pseudogenes were identified by CPGAVAS2 (Shi et al. 2019) and BLAST search, and tRNA genes were annotated by tRNAscan-SE (Schattner et al. 2005).

The chloroplast genome of *R. punjabensis* var. *sinica* (GenBank accession number: MT230555) was 159,617 bp long with 37.9% GC content. The GC content in IR regions, large-single copy (LSC) region, and small single-copy region (SSC) region were 43.0%, 36.0%, and 32.6%, respectively. The genome includes a LSC region of 87,694 bp, a SSC region of 18,971 bp, and a pair of inverted repeats of 26,476 bp. The genome contains 86 protein-coding genes, 8 rRNA genes, 2 pseudogenes, and 37 tRNA genes. In the protein-coding regions, the AT content of the third codon position (69.2%) was higher than that of the first (54.1%) and the second codon positions (61.8%).

The maximum-likelihood (ML) tree (Schattner et al. 2005) of *R. punjabensis* var. *sinica* and 14 species from order Sapindales based on complete chloroplast genome sequence, was constructed with *Dimocarpus longan* as outgroup (Figure 1). The ML tree with 1000 replicates revealed that the family Anacardiaceae was strongly supported as a monophyletic group and four species of *Rhus* were clustered into a clade. *Rhus punjabensis* var. *sinica* was sister to *R. potaninii* with 100% support value.

**Figure 1.**
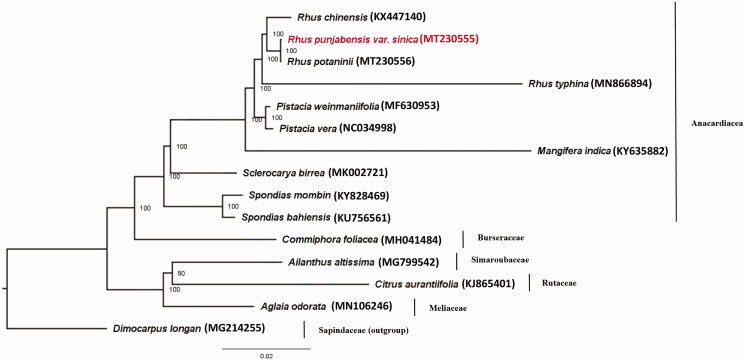
Phylogenetic tree of *Rhus punjabensis* var. *sinica* and fourteen species in order Sapindales using maximum likelihood (ML) analyses based on complete chloroplast genome sequences. The numbers at nodes of phylogenetic tree show the bootstrap support values.

## Data Availability

The genome sequence data that support the findings of this study are openly available in GenBank of NCBI at (https://www.ncbi.nlm.nih.gov/) under the accession No. MT230555. The associated BioProject, SRA, and Bio-Sample numbers are PRJNA688107, SRR13341544, and SAMN17169251, respectively.
